# Participation of Arachidonic Acid Metabolism in the Aortic Aneurysm Formation in Patients with Marfan Syndrome

**DOI:** 10.3389/fphys.2018.00077

**Published:** 2018-02-12

**Authors:** María E. Soto, Verónica Guarner-Lans, Karla Y. Herrera-Morales, Israel Pérez-Torres

**Affiliations:** ^1^Department of Immunology, Instituto Nacional de Cardiología “Ignacio Chávez”, Mexico City, Mexico; ^2^Department of Physiology, Instituto Nacional de Cardiología “Ignacio Chávez”, Mexico City, Mexico; ^3^Cardiothoracic Surgery, Instituto Nacional de Cardiología “Ignacio Chávez”, Mexico City, Mexico; ^4^Department of Pathology, Instituto Nacional de Cardiología “Ignacio Chávez”, Mexico City, Mexico

**Keywords:** aortic aneurysm, marfan syndrome, arachidonic acid, inflammation, cyclooxygenase

## Abstract

Marfan syndrome (MFS) is a pleiotropic genetic disease involving the cardiovascular system where a fibrillin-1 mutation is present. This mutation is associated with accelerated activation of transforming growth factor β (TGFβ1) which contributes to the formation of aneurysms in the root of the aorta. There is an imbalance in the synthesis of thromboxane A_2_ (TXA_2_) and prostacyclin, that is a consequence of a differential protein expression of the isoforms of cyclooxygenases (COXs), suggesting an alteration of arachidonic acid (AA) metabolism. The aim of this study was to analyze the participation of AA metabolism associated with inflammatory factors in the dilation and dissection of the aortic aneurysm in patients with MFS. A decrease in AA (*p* = 0.02), an increase in oleic acid (OA), TGFβ1, tumor necrosis factor alpha (TNFα), prostaglandin E_2_ (PGE_2_) (*p* < 0.05), and COXs activity (*p* = 0.002) was found. The expressions of phospholipase A_2_ (PLA_2_), cytochrome P450 (CYP450 4A), 5-lipoxygenase (5-LOX), COX2 and TXA2R (*p* < 0.05) showed a significant increase in the aortic aneurysm of patients with MFS compared to control subjects. COX1, 6-keto-prostaglandin 1 alpha (6-keto-PG_1α_) and 8-isoprostane did not show significant changes. Histological examination of the aortas showed an increase of cystic necrosis, elastic fibers and collagen in MFS. The results suggest that there are inflammatory factors coupled to genetic factors that predispose to aortic endothelial dysfunction in the aortic tissue of patients with MFS. There is a decrease in the percentage of AA, associated with an increase of PLA_2_, COX2/TXA2R, CYP450 4A, and 5-LOX which leads to a greater synthesis of PGE_2_ than of 6-keto-PGF_1α_, thus contributing to the formation of the aortic aneurysm. The evident loss of the homeostasis in these mechanisms confirms that there is a participation of the AA pathway in the aneurysm progression in MFS.

## Introduction

The Marfan syndrome (MFS) is a pleiotropic genetic disease with involvement of the cardiovascular, ocular and skeletal system with a very wide clinical variability. It is a connective tissue disorder with autosomal dominant associations in which the gene encoding for the fibrillin-1 (FBN-1) protein is affected (Wheeler et al., 2014).

FBN-1 is an essential protein in the formation and maturation of elastic fibers in the arteries and it is responsible for the assembly of the networks of microfibrils. The microfibrils store growth factors which are released at specific times to control growth and reparation of the tissues and organs of the body. Microfibrils and elastin form part of the extracellular matrix of tissues. FBN-1 is considered as a constitutive protein of connective tissue (Milewicz et al., 2008). The alteration in the expression of this protein can lead to destruction of the assembly of normal microfibrils and the production of abnormal elastic fibers and, as a consequence it leads to changes in elasticity in the aortic tissue causing growth and instability (Granata et al., 2016). This damage results in structural variations within the same arterial vessel with inherent heterogeneity in its content, thickness and cellular composition (Soto et al., 2014). In addition, defects or deficiencies in FBN-1 are associated with the regulation, bioavailability and accelerated activation of transforming growth factor β1 (TGFβ1) in the extracellular matrix. TGFβ1 contributes to the formation of aortic root aneurysms (Neptune et al., 2003). Also, the blockage of TGFβ1 release through anti-TGFβ1 antibodies reduces aortic root dilation in studies in mice (Cohn et al., 2007).

In addition, several non-genetic diseases are associated with aortic damage, and the etiology can therefore be multifactorial (Hendel et al., 2015). Oxidative stress(OS), the degree of inflammation and edema may contribute to the progression of aortic tissue damage associated with endothelial dysfunction (Soto et al., 2016a). In a preliminary study of our group, we evaluated the involvement of OS in different aortopathies in humans and correlated it with sub-endothelial basement membrane proteins and found the existence of dysfunction and progression of aortic damage (Soto et al., 2014). In another study, we showed that OS in MFS is associated with alterations in enzymes that employ glutathione, leading to increased chronic inflammation (Zúñiga-Muñoz et al., 2017). Furthermore, the vasomotor function in Marfan thoracic aorta is associated with an imbalance in the synthesis of thromboxane A_2_ (TXA_2_) and prostacyclin derived from the differential protein expression of cyclooxygenase (COXs) isoforms (Chung et al., 2007b). Levels of prostaglandin E_2_ (PGE_2_), TXA_2_, and IL-6 were significant higher in aortic aneurysms and were associate with overexpression in the aneurysmal wall of the inflammatory cyclooxygenase 2 (COX2) (Cheuk and Cheng, 2007).

On another hand, aortic damage and endothelial dysfunction with chronic inflammatory involvement have been associated to changes in arachidonic acid (AA) metabolism in several animal models and in cell lines both *in vivo* and *in vitro* (Aggarwal et al., 2012). Various physical and hormonal stimuli may stimulate the entrance of Ca^2+^ ions to the endothelial cell, increasing its intracellular concentration and activating the ion-dependent phospholipase A_2_ (PLA_2_) that releases the AA from the phospholipids of the cell membrane. Phospholipids can then be metabolized by the COXs isoforms, thromboxane synthase (TXs), lipoxygenase (LOXs) and cytochrome (CYP450) (Jamieson et al., 2017). The end products of these pathways are eicosanoids that can act in an autocrine and paracrine manner modulating vascular tone, platelet activation, signaling, proliferation, cell migration, fever and inflammatory processes. (Gauthier et al., 2011) Furthermore, AA can be non-enzymatically oxidized by free radicals, resulting in 8-isoprostanes which are oxidizing agents associated with OS and inflammation (Yang et al., 2010). Therefore, the evaluation of the participation of the AA metabolism is important since it may be involved in the development of aortic damage through activation of chronic inflammatory processes that lead to dilatation and dissection of the aortic aneurysm in MFS. The aim of this study was to analyze the participation of AA metabolism associated with inflammatory factors in the dilation and dissection of the aortic aneurysm in patients with MFS.

## Materials and methods

### Study design: the study was done on an observational cohort, and it is descriptive and prospective

#### Population in study

Patients with a Bentall and/or De Bono procedure (Bentall and De Bono, 1968) with aortic arch replacement, stent placement in the thoracic aorta, mitral valve replacement, mitral valvular change, coronary revascularization, Tirone David procedure and the procedure for the replacement of the thoracic-abdominal aorta were included. The following inclusion criteria were taken into account: aortic tissue was obtained from patient with a MFS diagnosis, based on the Ghent criteria. They had aortic dilatation and their cases had been presented in the surgical medical session to standardize the requirements of the type of aortic surgery to be considered according to the pathology of each patient. They were treated at the National Institute of Cardiology “Ignacio Chavez.” Exclusion criteria taken into account were a doubtful diagnosis and/or the lack of agreement to sign the informed consent form for the research study. Elimination Criteria were the insufficient tissue sample taken at the moment of surgery even when the patients met the inclusion criteria or the inappropriate conditions for the sample obtainment when considering the requirements for the research process.

#### Ethical considerations

The study was carried out according to the international ethical standards and the General Health Law, as well as according to the Helsinki declaration, modified at the Congress of Tokyo, Japan.

In all of the MFS patients and control subjects, studies of echocardiography, computerized tomography or magnetic resonance were performed. In the MFS patients, these studies were performed in order to determine the aortic lesion and valvular disease extension. In the control group, they were used to discard aortic damage additional to valvular damage. Therefore, only subjects who had isolated aortic stenosis without sclerosis and had no disease in the aorta were selected. Also, all patients selected had tricuspid aortic valve, patients with bicuspid valve were excluded. In the patients with MFS, dilatation of the aortic diameter (>50 mm), which is a criterion for the indication of surgery, was present and the risks and benefits of a surgical intervention were assessed in a clinical and pathological meeting with cardiovascular specialists. When patients were accepted for surgery, the preoperative protocol included coagulation tests, X-rays, electrocardiogram, anesthesia evaluation, and individualized medical intervention. Cases were dealt with caution, to avoid including patients undertaking treatment with antioxidants, allopurinol, or potential inhibitors of pathways involved in ROS production. Aspirin, warfarin, clopidogrel, and other antiplatelet or anticoagulant medications were suspended. In control subjects, routine laboratory tests were performed to determine acute phase reactants, triglycerides, and HDL cholesterol. None of the control subjects were taking anti-inflammatory drugs or statins. Control tissue was selected from patients that had an indication for surgery and in which aortic tissue could be obtained during the procedure that they required. The control subjects had trivalva aorta and underwent surgery for aortic stenosis. The surgery performed implied substitution of aortic valves, and the need to perform plastia or resection of aortic tissue surrounding the valvular area. During surgery, sample tissues of root thoracic aorta were obtained. Once the surgery was performed, the tissue was placed in liquid nitrogen and was kept at −30°C until used. The research protocol was approved by the Research and Ethics Committee of our institution (Institutional protocol number: 09654).

### Thoracic aorta homogenization

A sample from thoracic aorta was taken and placed in liquid N_2_ for homogenization. The homogenization process was previously described by Soto et al. (2014). Protein concentration in the thoracic aortic homogenate was determined by the method of Bradford (1976).

### Extraction and derivatization of the arachidonic and oleic acid

For AA and oleic acid (OA) extraction, 100 μg of protein from the aortic homogenates were used in the presence of 100 μg of nonadecanoic acid as internal standard and 2 ml of chloroform-methanol (2:1, vol/vol) with 0.002% BHT, as described by Folch (Folch et al., 1957) and previously report by López (López et al., 2016).

### Immunoblotting

One hundred microgram of protein from eachof the aortic homogenate were separated by SDS-PAGE (8% polyacrylamide gel) and transferred to a nitrocellulose Hybond-P membrane 45 μm (Millipore), according to methods previously described by Soto (Soto et al., 2014). The membranes were incubated overnight at 4°C with rabbit primary polyclonal anti-bodies against COX1(H-62, sc-7950), COX2 (H-62, sc-7951), PLA_2_(N-216, sc-438), TXA2R (H-120, sc-30036), 5-LOX (H-120, sc-20785) and mouse primary monoclonal antibody against CYP450 4A1/A2/A3 (clo4, sc-53247) (Santa Cruz Biotechnology, Santa Cruz, CA, USA).

### Cyclooxygenases activity assays

COXs activity was performed by monitoring the rate of O_2_ uptake using an oximeter (YSI oximeter model 5300A-1) which was coupled to an O_2_Clark type electrode. To 3 ml of a buffer of 0.1 M tris-HCl buffer, 1 mM phenol, 85 μg bovine hemoglobin, pH 8 and 100 μM AA at 37°C, 100 μg of aortic homogenate were added to initiate the reaction. Inhibition and discrimination of the catalytic activity of COXs was performed by the addition of 10 μM NS398 (COX2 specific inhibitor) and 1, 5, and 10 μM SC-560 (COX1 specific inhibitor). A unit of cyclooxygenase activity is defined as the ability of the enzyme to catalyze oxygenation of 1 nmol AA per minute at 37°C. The calibration curve was made with human COX2 (C0858-1000UN) provided by Sigma-Aldrich.

### Histology

The histological sections were processed according to conventional histological procedures and stained by methods previously described by Soto et al. (2014).

### Interleukins

6-keto-PGF_1α_, PGE_2_ and 8-isoprostane were provided by Cayman Chemical Company. TGFβ1 and TNFα were quantified by ELISA using commercial kits obtained by Elab science Biotechnology Co., Ltd. (Cat No. E-EL-ROO19) and Enzo Life Sciences (Cat No. ADI-900-155), respectively and read in a visible light microplate reader of 492/630 nm.

### Statistical analysis

For the analysis of continuous quantitative variables of normal distribution, t student test was used. For nonparametric data such as in general demographic characteristics Mann-Whitney *U*-test was employed. The program Sigma Plot version 11, Jandel Corporation was used to obtain the graphs. The data are presented as mean ± standard error. The differences were considered as statistically significant when *p* ≤ 0.05.

## Results

A total of 14 subjects with MFS and 6 controls were studied; the overall mean age was 40 ± 16 years. Age in patients with MFS had a median of 35 with a minimum value of 16 and a maximum of 59 and in controls it had a median of 63 with a minimum value 49 and maximum of 72 (*p* = 0.001), age and gender showed no differences. Demographic characteristics are shown in Tables 1, 2.

**Table 1:** General demographic characteristics of patients with MFS and subjects control.

	**Global**	**MFS**	**C**	***p***
**GENDER**				
F	11 (55)	10 (71)	1 (17)	0.03
M	9 (45)	4(29)	5 (83)	
**HEIGHT (m)**				
Median	1.70	1.70	1.60	0.02
(Min-Max)	(1.5–2.0)	(1.6–2.0)	(1.50–1.70)	
**WEIGHT (m)**				
Median	70	70	70	NS
(Min-Max)	(43–106)	(43–96)	(65–106)	
**BMI**				
Median	24	23	27	0.01
(Min-Max)	(17–37)	(17–33)	(24–37)	
**GLUCOSE (mg/dl)**				
Median	93	90	102	NS
(Min-Max)	(67–166)	(67–166)	(84–119)	
**LDL (mg/dl)**				
Median	95	96	101	NS
(Min-Max)	(64–154)	(64–154)	(70–125)	
**HDL (mg/dl)**				
Median	36	50	30	0.02
(Min-Max)	(13–62)	(23–62)	(13–36)	
**TRIGLYCERIDES (mg/dl)**				
Median	95	95	98	NS
(Min-Max)	(55–288)	(56–288)	(55–250)	

*F, Female; M, male; BMI, Body mass index; C, control; MFS, Marfan syndrome; n, number; HDL, cholesterol-high density lipoprotein; LDL, cholesterol low density lipoprotein. Global n = 20, MFS n = 14, and Control n = 6*.

**Table 2:** General demographic characteristics of patients with MFS.

**No**.	**Gender**	**Age**	**Criteria of the Ghent 2010 clinical nosology**				**Total number of Ghent criteria**
			**FH**	**Aortic diameter (mm)**	**Systemic score**	**Ocular**	
1	M	32	−	83	10	−	2
2	M	43	−	48	8	−	2
3	F	35	+	25	7	−	3
4	M	16	+	20.6	7	+	3
5	F	38	+	23	7	−	3
6	F	35	+	76	7	−	3
7	F	38	−	76	7	+	3
8	F	22	−	54	7	−	2
9	M	49	−	37	7	−	2
10	F	59	−	69	10	+	2
11	F	23	+	17	8	+	4
12	F	27	+	63	7	−	3
13	F	33	−	57	7	−	2
14	F	53	+	41	9	−	3

*MFS, Marfan syndrome; F, female; M, male; FH, family history*.

Table 3 shows the concentrations in pg/mg protein of the different markers measured. The 8-isoprostane AA oxidation marker was measured by a non-enzymatic assay. In patients with MFS it tended to increase but did not reach a statistically significant difference in comparison with the control subjects. The same tendency was observed in the 6-keto-PG_1α_ stable metabolite of COX1. However, PGE_2_, TGFβ1 and TNFα showed a significant increase in patients with MFS when compared to controls (*p* = 0.02, *p* = 0.04, *p* = 0.03, respectively).

**Table 3:** Inflammation, oxidative stress markers and vasodilator and vasoconstrictor prostaglandins products of the COX1 and COX2 respectively in control subjects and MFS.

**Parameters (pg/mg of protein)**	**Control**	**MFS**
8-isoprostane	15.6 ± 4.1	32.2 ± 8.5
6-keto-PG_1_α	2, 833.8 ± 893.9	3, 727.4 ± 696.8
PGE_2_	665.8 ± 101.6	**913.9** ± **49.3**^†^
TGF-β1	985.3 ± 230.6	**2,350.0** ± 372.5^*^
TNFα	169.2 ± 36.2	**349.2** ± 57.3^**^

MFS, Marfan syndrome. Control vs. MFS

† *p = 0.02*,

* *p = 0.04*,

** *p = 0.03*.

**Figure 1:**
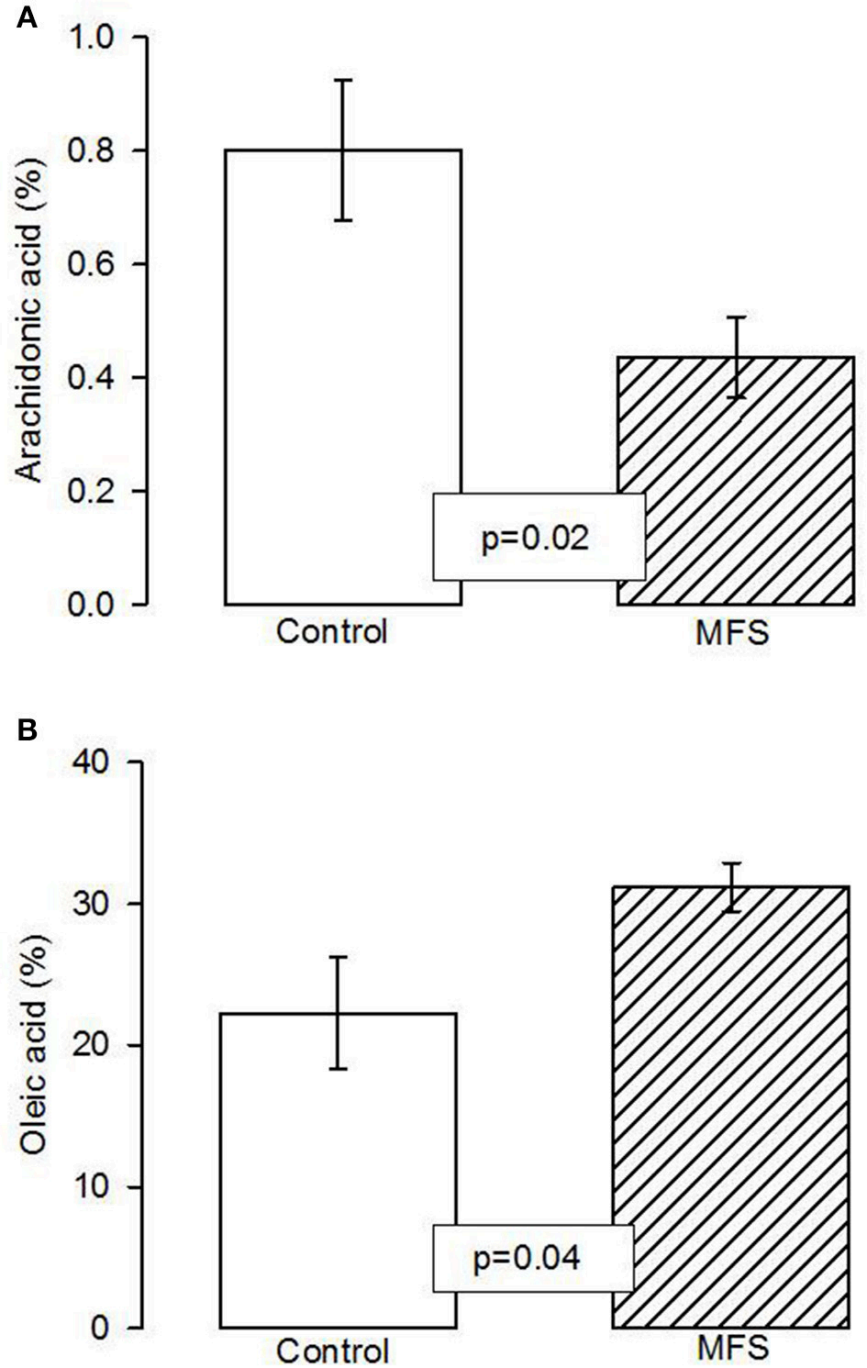
Total arachidonic acid percentage concentration **(A)** and of total oleic acid concentration **(B)** in the aortic aneurysm homogenate in control and MFS patients (*n* = 6; *n* = 14). The values are means ± SE. AA, Arachidonic acid; MFS, Marfan syndrome.

Figure 1A shows that the AA had a statistically significant percentage reduction in the aorta homogenate of MFS patients when compared with control subjects (*p* = 0.02). Figure 1B shows an increase of OAin MFS patients (*p* = 0.04) in comparison with control subjects.

**Figure 2:**
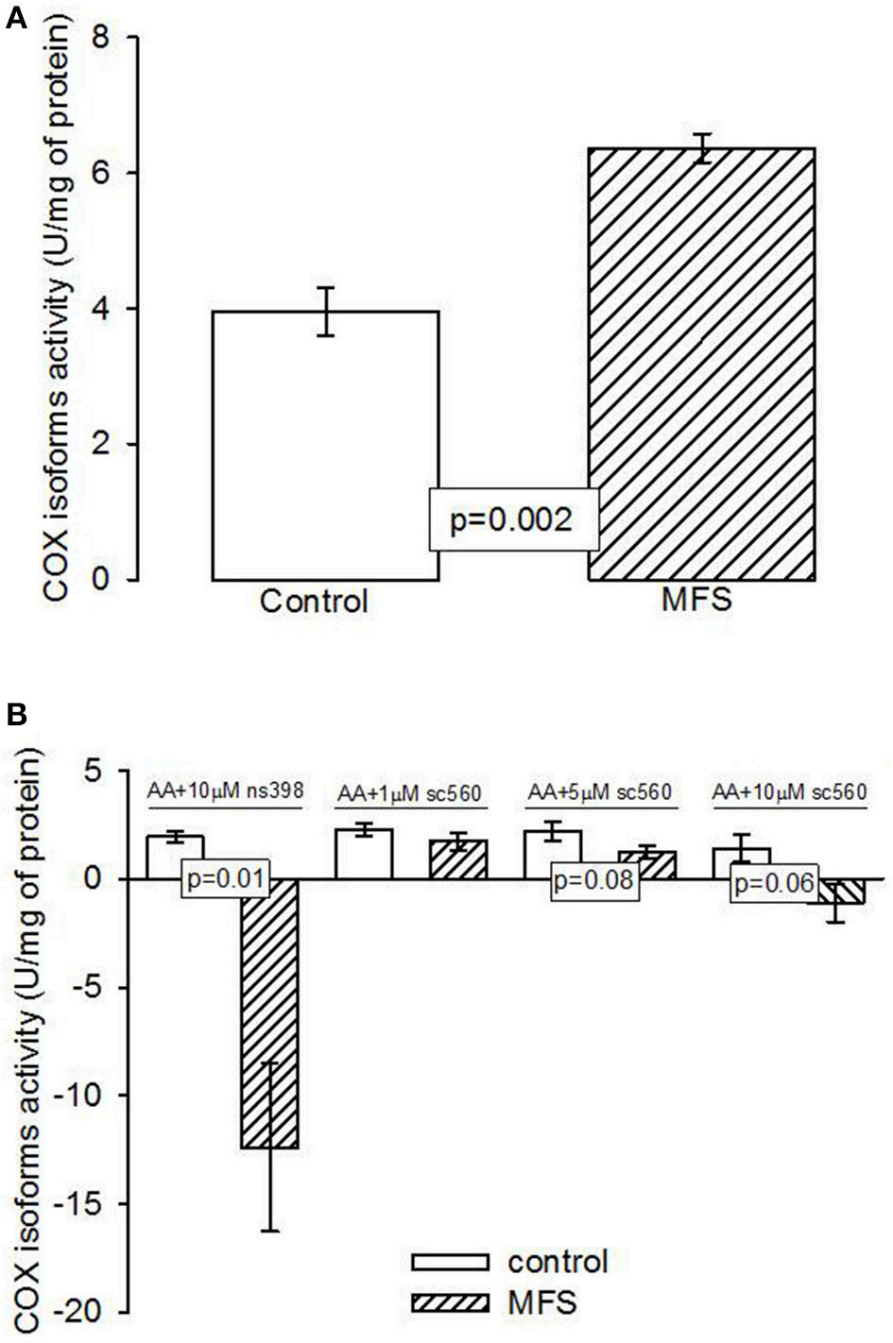
**(A)** Cyclooxigenases activity in the aneurysm of the aortic homogenate in control subjects (*n* = 6) and MFS patients (*n* = 14). **(B)** Inhibition of the cyclooxygenases in control subjects (*n* = 6) and MFS patients (*n* = 14). Data are means ± SE. COX, Cyclooxygenases; MFS, Marfan syndrome; NS398, specific inhibitor of cyclooxygenase 2; SC560, specific inhibitor of cyclooxygenase 1. The activity was determined by an oximeter coupled with a Clark type electrode and the time stroke was 7 min.

Figure 2A shows that the activity of the cyclooxygenase isoforms was statistically higher in patients with MFS compared to control subjects (*p* = 0.002). Figure 2B shows that inhibition of COX2 by NS398 was statically significant in patients with MFS compared to control subjects (*p* = 0.01). However, the inhibition of COX1 by SC560 in patients with MFS showed a tendency to decrease at concentrations of 5 and 10 μM (*p* = 0.08 and *p* = 0.06 respectively), but did not reach a statistically significant difference in comparison with the control subjects.

**Figure 3:**
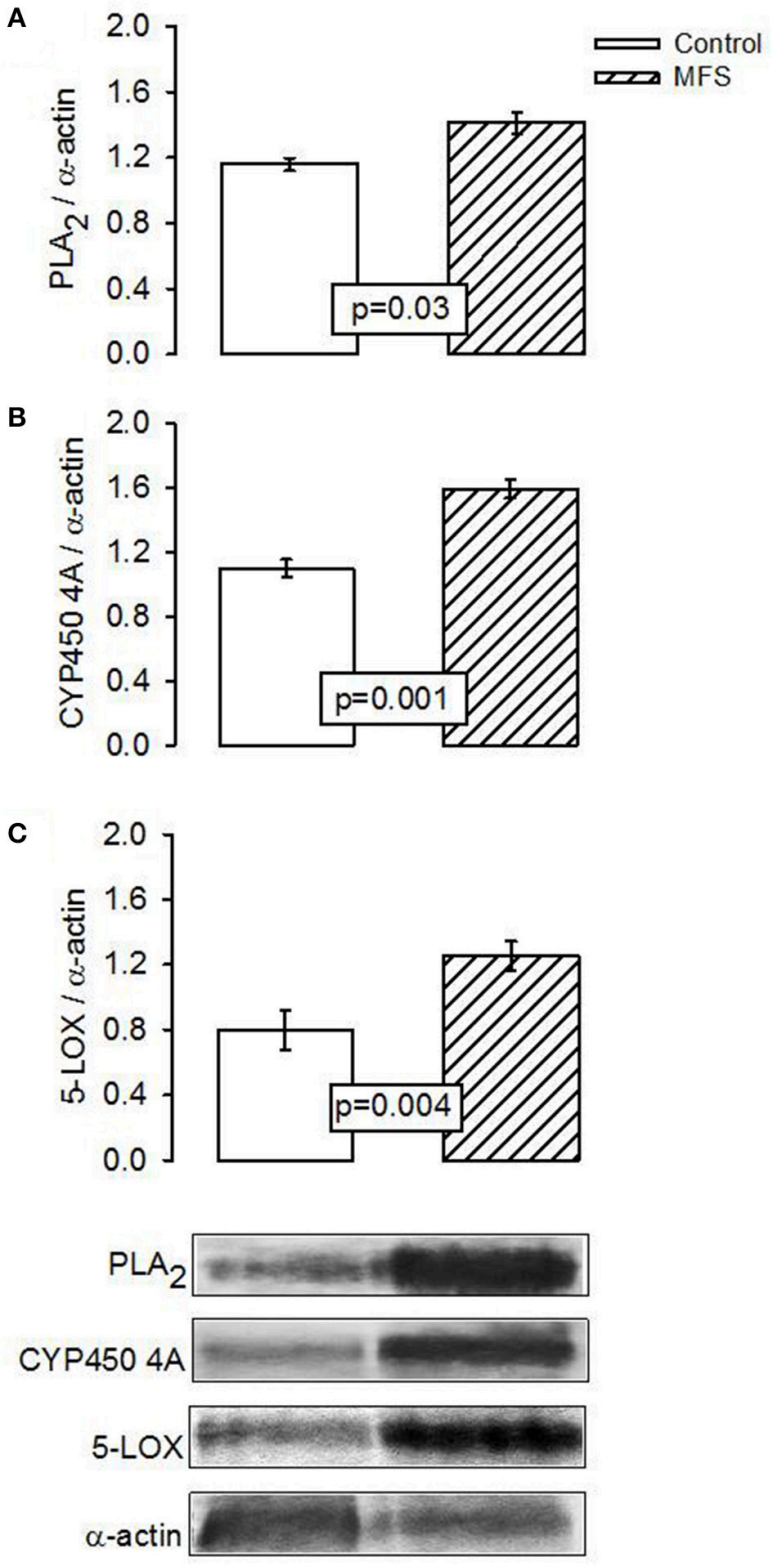
Representative image of the densitometric analysis of Western blots of **(A)** PLA2, **(B)** CYP450 4A, and **(C)** 5-LOX protein expressions in aortic aneurysm homogenate the control subjects (*n* = 6) and MFS patients (*n* = 14). Values are means ± SE.

In patients with MFS, the expression of PLA_2_, CYP450 4A, and 5-LOX showed a statistically significant elevation from that in control subjects (*p* = 0.03, *p* = 0.001, and *p* = 0.004, Figures 3A–C, respectively).

**Figure 4:**
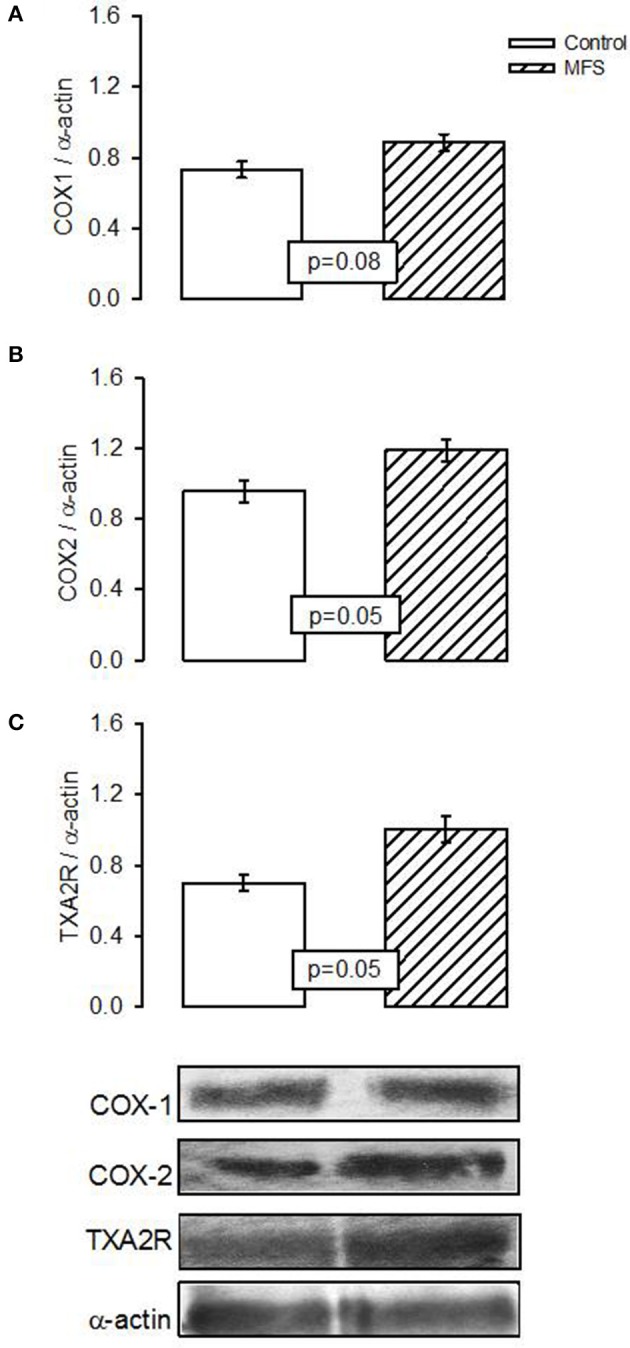
Representative image of the densitometric analysis of Western blots of **(A)** COX1, **(B)** COX2, and **(C)** Txs protein expressions in aortic homogenate of control subjects (*n* = 6) and MFS patients (*n* = 14). Data are means ± SE.

The expression of COX1 showed a tendency to increase in patients with MFS without reaching a statistically significant difference when compared to control subjects (*p* = 0.08, Figure 4A). However, expression of COX2 and TXA2R presented a significant increase in patients with MFS compared to control subjects (*p* = 0.05, Figures 4B,C, respectively).

**Figure 5:**
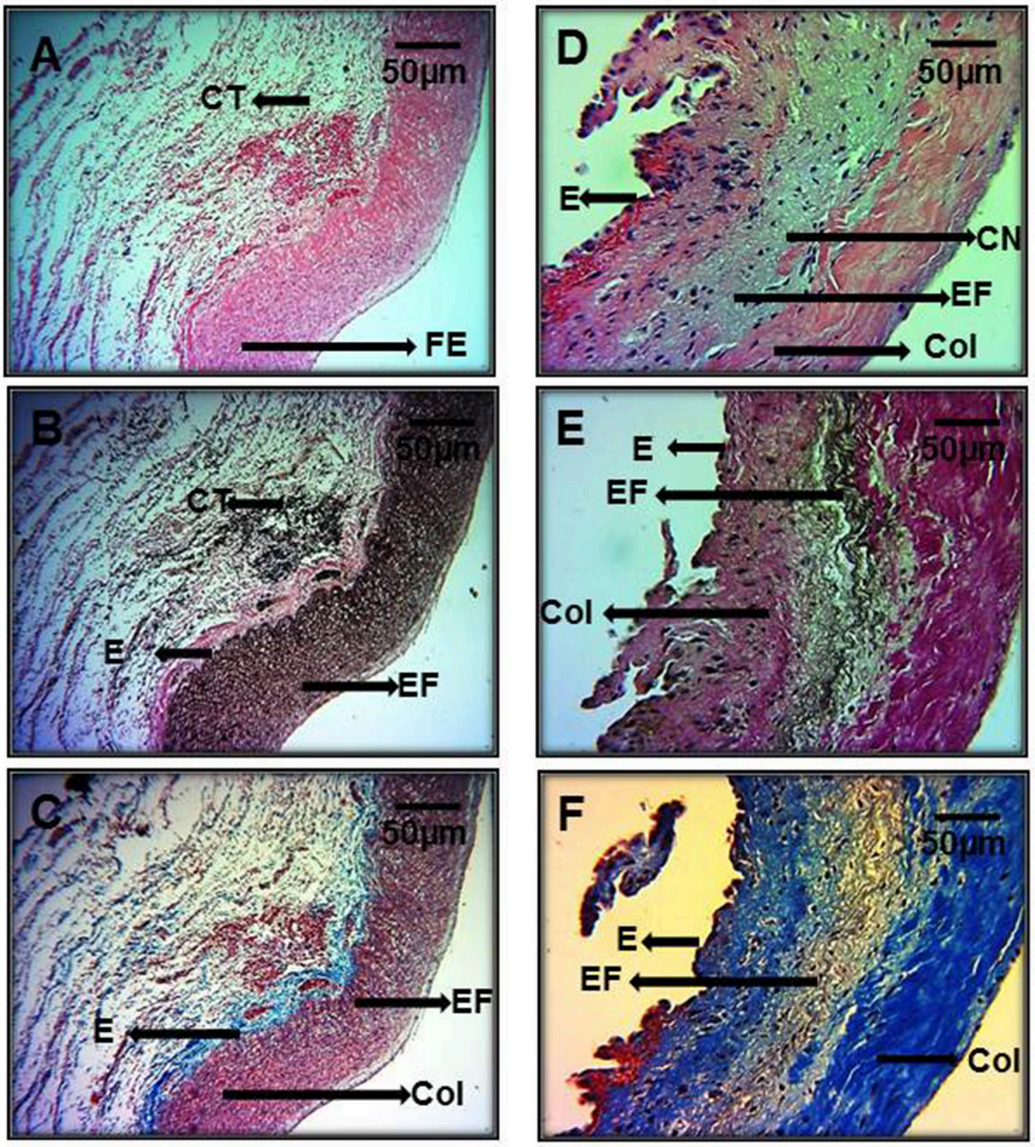
Representative photomicrographs of the aortic root in control subjects **(A–C)** vs. aortic root aneurysms in MFS patients **(D–F)**. Sections were stained with different histological techniques Hematoxylin-eosin, Masson's, and Weigert methods respectively. E, Endothelium; EF, elastic fibers; Col, collagen; CT, connective tissue; CN, cystic necrosis. 16x magnifications.

In Figures 5A–C representative photomicrographs of the hematoxylin-stained aorta, Masson's trichrome, and Weigers method for elastic fibers respectively of control subjects are shown. Elastic fibers shown in black can be seen alternating with limited collagen fibers in reddish brown. Figures 5D–F represent photomicrographs of the aortic tissue of patients with MFS respectively. There is an increase in collagen between broken elastic fibers that separate and form cavities resulting from the breakage of the elastic fibers. These characteristics correspond to the presence of cystic necrosis and suggest a lack of elasticity, thickening and high disorganization of the elastic lamellar structure and fibrosis due to excess collagen in patients with MFS.

**Figure 6:**
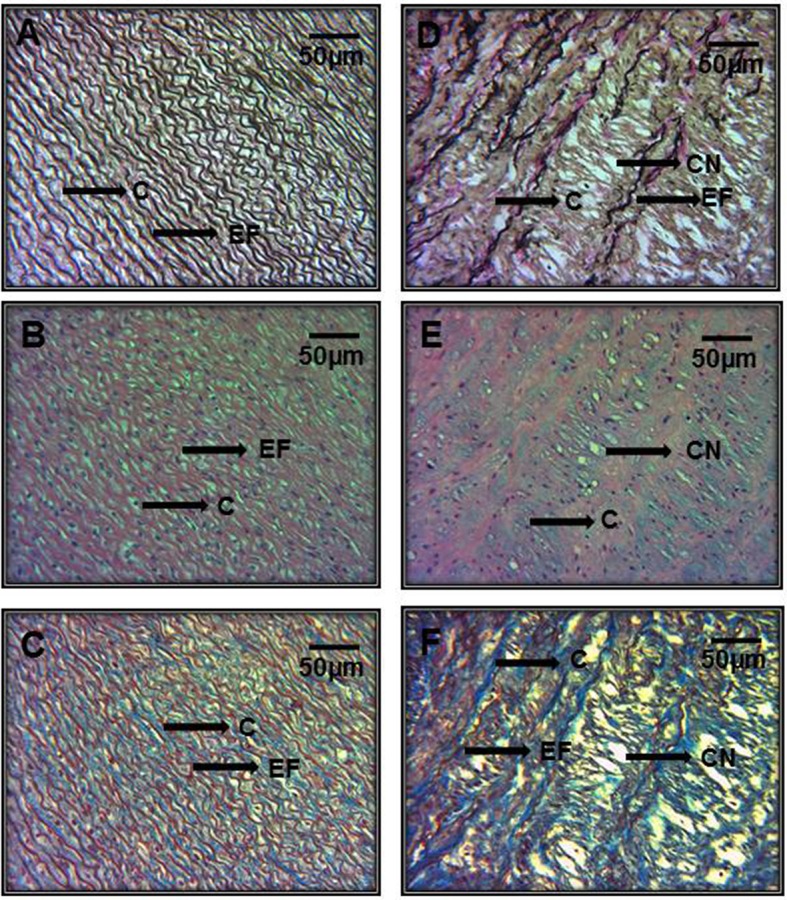
Representative photomicrographs of the aortic root at a 40x magnification. Aortic root aneurysms of control subjects **(A–C)** and of MFS patients **(D–F)**. Sections were stained with same histological techniques as in Figure 5. All MFS patients, had cystic necrosis, bundles of elastic fibers, thickening and rupture of elastic fibers, and increase in collagen between the elastic fibers in the aorta in comparison to C subjects. E, Endothelium; EF, elastic fibers; Col, collagen; CT, connective tissue; CN, cystic necrosis.

Figure 6 shows the same representative photomicrographs reported of control subjects in Figures 6A–C and of MFS patients in Figures 6D–F using the same histological techniques mentioned in Figure 5, but with a higher amplification of 40x. The histopathological changes are more evident than in Figure 5, where the magnifications were to 16x. These last pictures are panoramic and allow for the appreciation of part of the aortic vessel.

## Discussion

The aim of the study was to determine the participation of AA metabolism associated with inflammation factors in the aneurysm aortic dilatation and dissection in patients with MFS. AA is a major component of the membrane phospholipids necessary for the reparation and growth of skeletal muscle tissues. It is also a precursor of numerous eicosanoids (Hadley et al., 2016). The initiation of AA metabolism begins by the activation and translocation of the PLA_2_ and this enzyme releases AA from membrane phospholipids (Balsinde and Balboa, 2005). Different mechanisms can stimulate PLA_2_ such as ionophore A23187, increase in Ca^2+^ concentration through voltage dependent Ca^2+^ channels, activation of K^+^-ATP channels, stimulation of PKC (Linkous and Yazlovitskaya, 2010) and a pro-inflammatory state. The latter, in MFS may cause alterations in membrane lipid packaging and asymmetry which might increase PLA_2_. Our results show that PLA_2_ expression was increased in the aortic aneurysm of MFS patients. This increase suggests a high percentage of AA to be metabolized. However, the results show that AA decreases in MFS patients. This reduction can be, in part, attributed to the increase in OA since this acid can reduce the percentage of AA as has been described in serum and breast muscle (Haug et al., 2010). A recent study by our group described that OA is increased and involved in the development of aortic aneurysm in MFS patients. This study further shows a possible increase of the AA percentage without a reaching a significantly significant difference. However, in this new patient series, a statistically significant decrease of AA percentage was observed (Soto et al., 2016a). Although our results seem paradoxical, the AA reduction could depend on the degree of inflammation, aneurysm progression and the type of mutation present (Radonic et al., 2012).

The inverse relation between the percentage OA and AA could be caused by a positive feedback regulation, since a reduced percentage of AA could probably be expected if OA inhibits Δ^6^desaturase, elongase-5, and/or Δ^5^desaturase, the enzymes governing the formation of AA by the desaturation and elongation process from linoleic acid (Obukowicz et al., 1998; Høstmark and Haug, 2013). Furthermore, OA may induce the expression of COX2 (Fang et al., 2009), since an injection of OA increased prostanoids derived from COX2 in the pulmonary artery and was associated tothe presence of edema (Selig et al., 1987). Another possible explanation for the reduction of the percentage of AA may be its use as a substrate for the enzymes which metabolize it. This would lead to an increased expression and/or activity as is shown in our results. In addition, mice deficient in COX1, but not in COX2 exhibit a reduction in AA which contributes to edema and inflammation (Langenbach et al., 1995). This suggests that the AA percentage has to have the appropriate threshold since its broken equilibrium can cause adverse effects on the enzymatic pathways that depend on it.

On the other hand, our results show that the activity of COX isoforms is increased in MFS patients; however, this experiment does not allow for the discrimination of which of the two isoforms displays a greater participation. The immunoblot and the inhibition of the activity with the specific inhibitors show that COX2 expression and the inhibition of its activity were increased in MFS patients. However, the COX1 expression and inhibition also showed a tendency. This indicates that the enzyme that participates more is COX2, and it is therefore more important in the development of aortic aneurysm in MFS. Also, in ruptured abdominal aortic aneurysm, COX2 expression is significantly elevated (Chung et al., 2007b). Another study showed an increase in the COX2 expression in the thoracic aorta of the Fbn1^C1039G/+MFS^ mouse model, while COX1 was down-regulated (Chung et al., 2007a).

PGE_2_ biosynthesis by COX2 is important for many biological functions and is generally very low in un-inflamed tissues, but increases immediately in acute inflammation prior to the recruitment of leukocytes and infiltration of immune cells (Ricciotti and FitzGerald, 2011). Under these physiological conditions, PGE_2_ can modulate various steps of inflammation through receptors such as those for IL-1β, IL-6, and MCP-1 (Babaev et al., 2008). In wild-type mice, the deficiency of COX2 reduces the level of PGE_2_ production by approximately 75%, while the deficiency of COX1 reduces the PGE_2_ level by 25%. This indicates that both COX isoforms contribute to PG production during inflammation and also that COX2 derived PGs appear to be more important in both the acute inflammatory process and in the resolution phase (Langenbach et al., 1999). Our results show an increase in PGE_2_ in comparison to 6-keto-PG_1α_ in aortic aneurysm tissue from MFS patients that were associated with increase and decrease of COX2 and COX1 respectively. This indicates a high contribution of PGE_2_ in the aortic aneurism formation in MFS. Furthermore, PGE_2_ biosynthesis by COX2 is increased in human abdominal aortic aneurysm and the infiltration of leukocytes in the aortic wall and may potentially contribute to the PGE_2_ increase (Solà-Villà et al., 2015). In addition, PGI_2_ is a potent vasodilator and inhibitor of platelet aggregation, leukocyte adhesion and it increases the production of the anti-inflammatory cytokine IL-10 (Francois et al., 2005), and is rapidly converted by non-enzymatic processes to an inactive hydrolysis product, 6-keto-PGF_1α_ (Wu and Liou, 2005). Additionally, PGI_2_ is more abundant in the aorta than PGE_2_ (Qi et al., 2006). However, when COX1 is inhibited PGI_2_ is significantly decreased while PGE_2_ is increased in the aorta by COX2 overexpression, and the inflammatory chronic process in MFS can contribute to this (Qi et al., 2006).

Additionally, the limiting step in the synthesis of TXA_2_ is COX2/TXs enzyme on AA, and the increase of this enzyme has been associated with a TXA_2_ increase (Jiang et al., 2007). In the thoracic aortic dissection from Marfan mice at 3, 6, and 9 months of age that were heterozygous for the Fbn^1C1039g/+^ allele, COX1 expression was down-regulated, COX2 was increased and there was an imbalance in the synthesis of TXA_2_ which was associated with vascular hyperplasia, thrombosis events and vascular remodeling (Chung et al., 2007a). Our results show that the TXA2R expression was increased in aneurysm tissue from MFS patients. This indicates that TXA_2_ synthesized by COX2/TXs enzyme can increase TXA2R and contribute to the progression of aortic aneurism in MFS patients (Chung et al., 2007b). In addition, TXA_2_ produced by COX2 promotes inflammatory mediator production that participates in vascular injury, hypertrophy, platelet aggregation and extracellular matrix formation. Thus, it may be a factor influencing the expansion rate and eventual rupture of the aneurysm (Cheuk and Cheng, 2007). Therefore, our results suggest that, in the aorta the MFS patients, there exists an imbalance in the synthesis of the prostaglandins given by an increase of PGE_2_, TXA_2_ and decrease of 6-keto-PG_1α_ which result from the altered metabolism of AA through the rate limiting enzymes COX1 and COX2. This imbalance contributes in part to the compromised aortic vasomotor function in MFS, impairing the release of endothelial relaxant molecules (Di Marzo, 1995; Soto et al., 2016a).

On the other hand, PGE_2_ and TXA_2_ increase transcription of type IV collagen, laminin and fibronectin. These proteins are involved in the thickening of the aortic sub-endothelial layer of the aortic aneurysm (Pricci et al., 1996). The increases in PGE_2_ and TXA_2_, participate in the alteration of the contraction and relaxation of vascular smooth muscle, together with the inflammatory chronic processes, which influence metalloproteinase (MMPs) expression (Dorn et al., 1992). Furthermore COX2 over-expression induces MMPs whose activation can result in the extracellular matrix degradation that is essential for vascular remodeling and inflammatory cell infiltration that contribute to the formation of the aortic aneurysm (Gitlin et al., 2007). In addition, the chronic inflammatory disease present in the aortic aneurysm of MFS is produced by the FBN-1 mutation (Holm et al., 2011). This FBN-1 mutation could easily affect the organization of the collagen fibers in the aortic adventitia through an increase in TGFβ1 signaling that occurs in the extracellular matrix and contributes to increased collagen production (Holm et al., 2011). These changes lead to aortic damage due to impaired resistance to pressure, and by creating a positive feedback that may cause collagen damage. The degradation products resulting from this process may induce chronic inflammation in MFS patients (Radonic et al., 2012). Furthermore, TGFβ1 can also regulate the over expression of elastase and many MMPs such as -2 and -9 (Neptune et al., 2003). Also, an increased level of elastase might increase elastin degradation mediated by the MMPs, and can be responsible for the disintegration of elastic fibers that reduce connective tissue elasticity and lead to weakness of the aortic wall (Benke et al., 2013). Besides, the soluble peptide fragments derived from the degradation of extracellular matrix components, including elastin, laminin and fibronectin may also serve as chemotactic agents for infiltration by macrophages which are responsible for the enhanced release of inflammatory mediators (Cheuk and Cheng, 2008). However, hemodynamic forces, trans-mural inflammation, imbalance of MMPs, increase of TGFβ1, inflammatory cell infiltrates, apoptosis, ROS, fatty acid alteration and interleukins such as TNFα also participate (Soto et al., 2016a).

Our results show that TGFβ1 and TNFα concentrations in the homogenized tissue from the aortic aneurysm the MFS patients were increased, and this increase was associated with accumulation of collagen, cystic necrosis and degradation of elastic fibers as observed in the photomicrographs. A study shows that indomethacin, an unspecific inhibitor of COXs isoforms, significantly improves elastin integrity and reduces the numbers of macrophages in the adventitia of heterozygous mgR/mgR FBN1 in a Marfan mice model. These changes coincide with decreased MMP-2, -9, and -12 expressions, and are blocked by a decrease in the TGFβ1 activity in the aorta, and with improved elastic lamellae architecture. These changes were associated with a decrease in COX2 expression and PGE_2_ concentration (Guo et al., 2013). In addition, an alteration in the AA percentage has been associated with increased production and secretion of TNFα (Cubero and Nieto, 2012). TNFα can induce a switch from the PGD_2_ to the PGE_2_ synthesis pathway by regulating PGE_2_ synthase expression and/or activity. It can also act on activators of PKA that potentiate markedly the TNFα-induced increase in PGE_2_ through up-regulation of COX2 gene expression (Fournier et al., 1997). In a study using pre- to post-operative arterial blood samples for 13 days in patients with ruptured aneurysms and intact aneurysms, an increase of TNFα, IL-1β, and IL-6, was found (Swartbol et al., 2001). Also, TNFα derived from intrinsic vessel wall components or the cells of the tymphomonocytic infiltrate, is part of an accelerated proteolytic cascade that is responsible for progressive destruction of structural matrix proteins, particularly collagen and elastin. This destruction leads to a thin degraded media layer with significant loss of the elastic component and a fibrotic and or inflammatory adventitia (Fernandez-Moure et al., 2011). TNFα is also involved in the early phase of the cytokine cascade in a pro-inflammatory state that promotes endothelial dysfunction and induction of pro-inflammatory genes including those of iNOS and COX2 (Lu et al., 2015). TNFα may stimulate endothelial cells to express vascular cell adhesion molecule-1, intracellular- adhesion molecule-1 which facilitates macrophages infiltration with the secretion of collagen, elastin and proteoglycans to form a fibrous matrix resulting in the changes in the aortic wall architecture (Grötzinger, 2002). Similarly, the excess release of TGFβ1 from the connective tissue of the matrix can cause an increased activation of the smooth muscle cells and a haphazard and inappropriate remodeling response, which is characterized by excess deposition of matrix elements such as collagen, proteoglycans, MMPs-2, -9 and infiltration of macrophages (Zhang et al., 2013).

The results of TNFα and TGFβ1 determinations obtained in this study are associated with the thickening and high disorganization of the structure of elastic lamella in the aortic aneurysm of MFS patients. This alteration of the aortic medial layer can generate cystic necrosis and fibrosis by collagen excess in MFS (Guo et al., 2006). This contributes to the reduced elasticity and compliance in the aortic aneurysm, the loss of elastic fiber integrity and the increase in stiffness which result in a significantly higher breaking stress in the aorta the MFS patients (Yuan and Jing, 2011). Besides, a recent paper where a histopathological analysis in MFS patients was performed, showed an increase MMP-9, angiotensin II and TGF-β1 productions which was associated with cytolytic necrosis and elastic fiber degeneration (Grewal et al., 2016). In a similar manner, high concentrations of TGFβ1 and TNFα, favor apoptosis in endothelial cells and activate MMPs-1 and -2 in vascular smooth muscle cells (Ramachandra et al., 2015). It therefore promotes collagen and elastin degradation in the extracellular matrix, which contributes to physically inhibit the normal dilatation of the aorta. MMP-2 has also been associated with the development of aneurysms in the thoracic aorta (Ramachandra et al., 2015). Our results showed that all MFS patients had cystic necrosis and an increase of collagen and elastic fibers rupture.

On another hand, AA can also be metabolized through other pathways, such as 5-, 12-, 15-LOX, and CYP450 pathway (Funk, 2001; Ferrucci et al., 2006). Our results show that CYP450 4A and 5-LOX expressions were increased in the aortic aneurysm of MFS patients. These results suggest that other enzymes involved in AA metabolism also contribute to the aortic aneurysm progression in MFS, and provide another possible explanation to the contribution of the AA reduction. This could be related to an increase in use AA by CYP450 4A and 5-LOX enzymes, which can contribute to aneurysm progression in MFS. Hence, AA has an anti-thrombogenic effect via CYP450 generated EETs; this eicosanoids possess vasodilator properties, and may inhibit the COX2 (Krötz et al., 2004). This suggests that the CYP450 4A overexpression can be a compensatory mechanism to counteract the other pathways of AA metabolism that are altered. However, this pathway is unable to completely counteract the other effects of the AA metabolism. In contrast, various components of the 5-LOX pathway are involved in human vascular disease (Cao et al., 2007). In Apoe^−/−^ mice with an atherosclerotic producing diet, 5-LOX in the lamina adventitia rather than in the lamina intima contributed to the formation of aortic aneurysms associated with production of systemic inflammatory leukotrienes that indirectly affected extracellular matrix components including MMP-2 (Zhao et al., 2004). A study in advanced lesions in human coronary arteries showed that there is abundant 5-LOX in the vascular adventitia (Spanbroek et al., 2003). Another study showed that leukotriene via 5-LOX inhibited the production of PGI_2_ by COX1 in the vascular endothelium and indirectly contributed to overall vascular constriction (Chawengsub et al., 2009).

Furthermore, OS is associated with endothelial dysfunction in a mouse model and in MFS patients. It contributes to increases the aneurysm formation (Zúñiga-Muñoz et al., 2017). OS is associated with decreased nitric oxide and PGI_2_ mediated endothelium-dependent relaxation via eNOS and COX1. Furthermore, OS is associated with the aortic structural changes that lead in part to the formation of aneurysms (Soto et al., 2016b; Zúñiga-Muñoz et al., 2017). Significantly increased levels of isoprostane 8-epi-PGF2α have been recognized as oxidative markers associated with vascular diseases such as vascular reperfusion, and have also been found in plasma and aortic homogenate of MFS mice. However, 8-isoprostane is also a product of the AA metabolism which is oxidized by free radicals and by the non-enzymatic pathway (Łuczaj et al., 2015). The results in the homogenized tissue from the aortic aneurism from MFS patients showed a tendency of 8-isoprostane to increase without reaching a significant value (Yang et al., 2010). This indicates that high concentrations of this product of oxidation, could favor endothelial cell apoptosis and activate MMPs-1 and -2 in vascular smooth muscle cells. It therefore promotes the degradation of various components of the extracellular matrix such as collagen and elastin (Fujiwara et al., 2008). This could, in part, contribute to progression in the aortic aneurysm in MFS patients.

## Conclusion

In the aortic tissue of MFS patients there are inflammatory factors coupled with the genetic factors that predispose to aortic endothelial dysfunction. There is a decrease in the percentage of AA, which is associated with an increase of PLA_2_, COX2/TXs, CYP450 4A and 5-LOX which leads to a greater synthesis of PGE_2_ than 6-keto-PGF_1α_ thus contributing to the formation of the aortic aneurysm. The evident loss of homeostasis in these mechanisms confirms that there is a participation of the AA pathway associated to the degree of inflammation and the aneurysm progression in MFS.

### Study limitations

The use of aortas from MFS patients and C subjects constitutes an important limitation of this study. The obtainment of tissue from aortic samples is very difficult despite the informed consent of MFS patients and control subjects and the aortic sample size is very small. The incidence MFS is of 2-3 per 10,000 individuals, being an autosomal dominant disorder of the connective tissue caused by mutations. The monitoring of each patient prospectively for a long time is also practically impossible. Retrospective studies only allow for the evaluation of some aspects but do not give the opportunity to correct some biases. The results from this study suggest the importance of studying the general inflammatory profile in this disease to correlate it to the local inflammation in the aortic tissue in future studies. Another limitation to this study is the improbability of having matched controls for age and gender, since it is not possible to obtain tissue from healthy people. The only way to obtain the tissue is from subjects having a surgical indication where there is a possibility to ethically withdraw a small sample. This depends on the surgical technique of the treatment applied to the subjects and the informed consent of the patients. However, even though there are age differences in controls and patients, there is certainty that there were no co-morbidities or aortic damage as shown by the imaging studies reported.

## Author contributions

MS: designed the study, diagnosed the patients and designed the tables. VG-L: wrote, restructured and reviewed the manuscript. KH-M: obtaining the sample, IP-T: designed the study, wrote the manuscript, performed the assays and interpreted the results.

### Conflict of interest statement

The authors declare that the research was conducted in the absence of any commercial or financial relationships that could be construed as a potential conflict of interest.
